# The TELCoMB Protocol for High-Sensitivity Detection of ARG-MGE Colocalizations in Complex Microbial Communities

**DOI:** 10.1002/cpz1.70031

**Published:** 2024-10

**Authors:** Jonathan E. Bravo, Ilya Slizovskiy, Nathalie Bonin, Marco Oliva, Noelle Noyes, Christina Boucher

**Affiliations:** 1 Department of Computer and Information Science and Engineering, Herbert Wertheim College of Engineering, University of Florida, Gainesville, Florida; 2 Department of Veterinary Clinical Sciences, College of Veterinary Medicine, Purdue University, West Lafayette, Indiana; 3 Purdue Applied Microbiome Sciences Program, Purdue University, West Lafayette, Indiana; 4 Department of Veterinary Population Medicine, University of Minnesota, St. Paul, Minnesota; 5 Department of Computer Science, College of Engineering, University of Maryland, College Park, Maryland

**Keywords:** antimicrobial resistance, bioinformatics, colocalizations, megares, microbiology, snakemake

## Abstract

Understanding the genetic basis of antimicrobial resistance is crucial for developing effective mitigation strategies. One necessary step is to identify the antimicrobial resistance genes (ARGs) within a microbial population, referred to as the resistome, as well as the mobile genetic elements (MGEs) harboring ARGs. Although shotgun metagenomics has been successful in detecting ARGs and MGEs within a microbiome, it is limited by low sensitivity. Enrichment using cRNA biotinylated probes has been applied to address this limitation, enhancing the detection of rare ARGs and MGEs, especially when combined with long-read sequencing. Here, we present the TELCoMB protocol, a Snakemake workflow that elucidates resistome and mobilome composition and diversity and uncovers ARG-MGE colocalizations. The protocol supports both short- and long-read sequencing and does not require enrichment, making it versatile for various genomic data types. TELCoMB generates publication-ready figures and CSV files for comprehensive analysis, improving our understanding of antimicrobial resistance mechanisms and spread.

**Basic Protocol 1:** Installing TELCOMB Locally

**Alternate Protocol:** Installing TELCOMB on a SLURM Cluster

**Basic Protocol 2:** Data Preprocessing

**Basic Protocol 3:** Calculation of Resistome Distribution and Composition

**Basic Protocol 4:** Identification of ARG-MGE Colocalizations

## INTRODUCTION

Understanding the genetic basis of antimicrobial resistance is crucial for developing strategies to mitigate its impact. Efforts to combat resistance include prudent use of antimicrobial agents (Anthony et al., 2001; Lutz et al., 2020), development of new drugs (Cook & Wright, 2022; Tarín-Pelló et al., 2022), and implementation of infection prevention and control measures to reduce the selective pressure favoring the survival of resistant microorganisms (Chinemerem Nwobodo et al., 2022). Antimicrobial resistance genes (ARGs) are genetic elements that encode mechanisms that allow microorganisms to withstand the effects of antimicrobial agents. Accurately identifying all ARGs within a microbial sample, referred to as the resistome, is vital to the study of antimicrobial resistance because it allows the characterization of all potential mechanisms and classes of resistance within a given microbial population. Moreover, understanding the diversity and abundance of mobile genetic elements (MGEs) in a microbial population, which is referred to as the mobilome, is equally important given their role in horizontal gene transfer, influencing microbial evolution, adaptation, and the spread of ARGs, thereby impacting both environmental and clinical ecosystems. In the past several years, shotgun metagenomics has proven to be successful in mapping the ARGs and MGEs present in various microbial communities and environments. Due to this success, shotgun metagenomics has become a common approach for understanding the microbial environment in clinical, agricultural, and ecological contexts. Despite its advantages, shotgun metagenomics grapples with challenges related to relatively low sensitivity (Noyes et al., 2016; Rose et al., 2016), which reduces our ability to identify ARGs and/or MGEs that can have low abundance within a microbial community. Indeed, prior studies demonstrated that the percentage of sequence data compromising ARGs from shotgun metagenomics is often less than 1 % of the data, and clinically relevant ARGs are often even less abundant (Carr et al., 2021; Noyes et al., 2016; Zaheer et al., 2018).

To overcome the lack of sensitivity for the detection of (rare) ARGs and MGEs, enrichment using cRNA biotinylated probes has been successfully applied to metagenomic samples, allowing for the recovery of these very rare ARGs and MGEs. This probe-based enrichment is becoming increasingly common, especially since it can be combined with both short- and long-read sequencing (Slizovskiy et al., 2020). The ability to combine cRNA biotinylated probes with long-read sequencing is particularly beneficial because it enables the unambiguous identification of ARG-MGE colocalizations, i.e., single reads that contain both an ARG and an MGE. One of the concerning aspects of antimicrobial resistance is the ability of ARGs to spread horizontally between bacteria, even across species. Horizontal gene transfer can occur through conjugation, transformation, and transduction, allowing ARGs to move between bacteria and contribute to the development of resistant strains. Elucidating the MGEs that occur in conjunction with ARGs is vital to gaining a comprehensive view of the risk posed by ARGs and their ability to be transferred via MGEs. However, there is currently no effective bioinformatic method for identifying ARG-MGE colocalizations effectively within metagenomic data.

**Figure 1:**
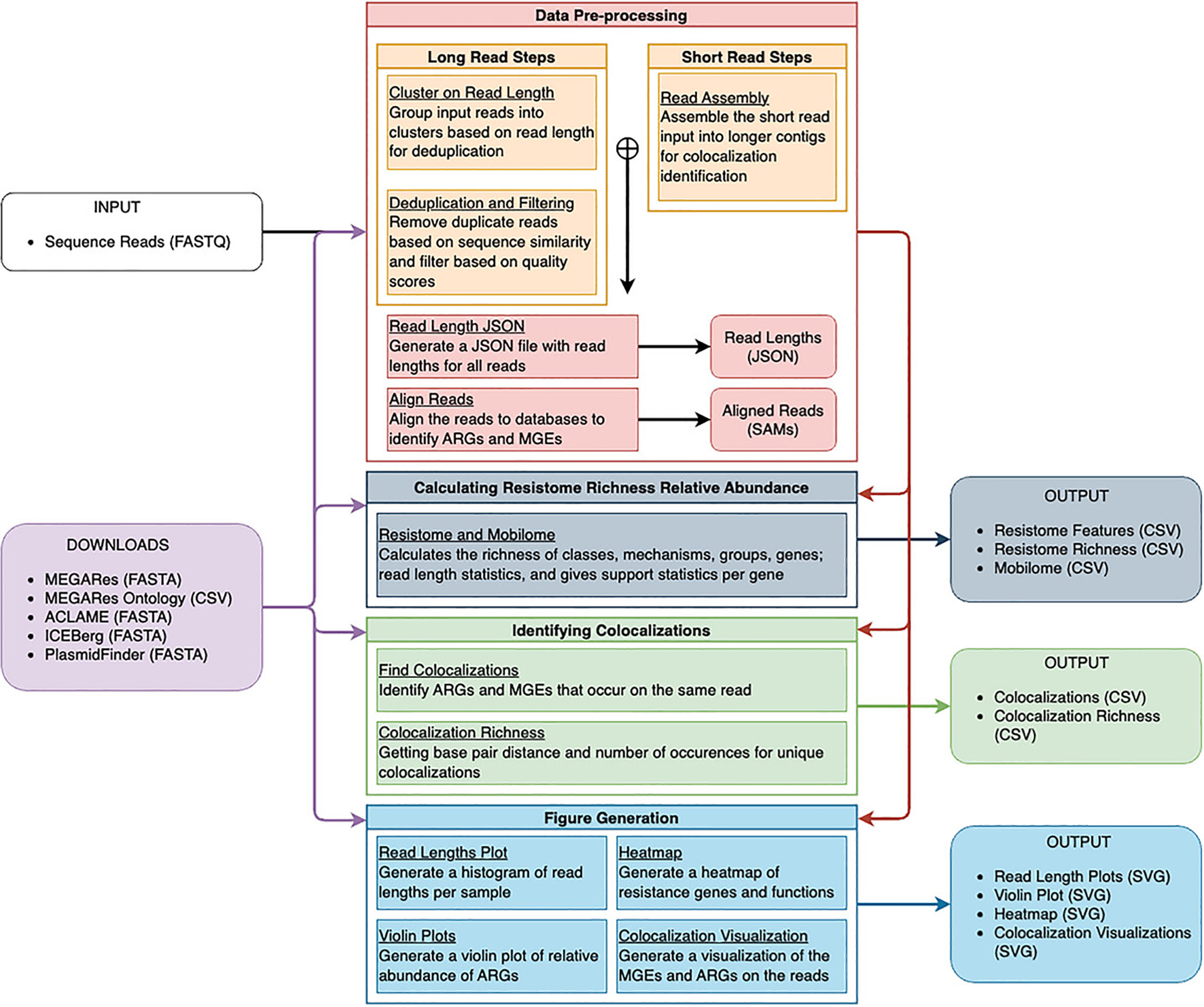
TELCoMB Flow. The TELCoMB workflow consists of four groups: (1) data preprocessing, (2) resistome richness and relative abundance calculations, (3) colocalization identification, and (4) figure generation. Data preprocessing steps depend on the sequencing technology: long reads are clustered and deduplicated, while short reads are assembled using MetaSPAdes. A json file of read lengths is created for later processing, and the reads are aligned to identify MGEs and ARGs. Calculating resistome richness and relative abundance involves a rule that calculates richness and feature counts for both the resistome and mobilome. Colocalization identification involves two rules: finding ARGs and MGEs on the same read and calculating colocalization statistics. Figure generation includes four rules: plotting read lengths, a violin plot of ARG relative abundance, a presence/absence heatmap for ARGs, and a colocalization figure per sample.

Here, we present a detailed description of the TELCoMB protocol, which analyzes metagenomic data to generate comprehensive resistome, mobilome, and ARG-MGE profiles. TELCoMB is implemented as a Snakemake workflow (Mölder et al., 2021) and is publicly available on GitHub at https://github.com/jonathan-bravo/TELCoMB under the GNU license. Figure 1 gives an overview of the workflow. Although it was developed primarily to function with target-enriched long-read sequencing data, it can also handle short-read data and is agnostic to long-read technology (i.e., Oxford Nanopore Technologies or PacBio). When used with short reads, the reads are assembled to create contigs in an attempt to recreate the same genetic resolution found in the long read sequencing. Additionally, the protocol does not require enrichment and can take as input unenriched data. Hence, TELCoMB is able to successfully work on almost all genomic data types. TELCoMB provides a variety of publication-ready figures and CSV files that allow for downstream analysis of resistome and mobilome composition and diversity, and ARG-MGE colocalizations. This approach not only improves our ability to study the resistome and mobilome but also provides critical insights into the mechanisms driving antimicrobial resistance and its spread within microbial communities, which can be used to inform public health strategies and address antimicrobial resistance more effectively.

## BASIC PROTOCOL 1

### INSTALLING TELCOMB LOCALLY

#### Necessary Resources

##### Hardware

TELCoMB can be run on a variety of computer hardware. We recommend a minimum of 16 CPU cores and 32 GB of RAM, even though the workflow can be run on less; for any samples larger than a couple of megabytes, the workflow will be limited in its ability to run rules in parallel. Disk space required will be determined by the amount of input data, but you can expect roughly 700 MB of output data for every 165 MB input FASTQ file provided; plus roughly 12 GB of temporary data, work_dir/tmp/*, which can be removed after the workflow has completed running. We also recommend roughly 200 MB of storage for the databases that are downloaded as a part of the workflow.

##### Files

TELCoMB requires the user to specify the directory containing the sequence data. A number of reference files are also downloaded and used for analysis.

- **Sequence reads:** the sequenced(s) read in FASTQ format; a single file per sample for long-read input, or a pair of files with the form sample_name_{R1,R2}.fastq for short-read input
- **MEGARes database:** the MEGARes database and ontology are downloaded as a FASTA file and a CSV file, respectively. MEGARes is needed because its ontology is used by TELCoMB. The CSV file contains annotations of the MEGARes ARGs. The file format of the CSV file is as follows:
  - Column 1: read header matching an accession header in the FASTA file
  - Column 2: the type of antimicrobial compound to which the accessions confer resistance
  - Column 3: the major antimicrobial chemical class to which the accession confers resistance
  - Column 4: the biological mechanism of resistance by which the accession confers resistance
  - Column 5: the gene- or operon-level group for the accession
- **ICEberg database:** a FASTA file containing the sequences in the ICEberg database is downloaded.
- **ACLAME database:** a FASTA file containing the sequences in the ACLAME database is downloaded.
- **PlasmidFinder database:** a FASTA file containing the sequences in the PlasmidFinder database is downloaded.

##### Software

We note that all dependencies are managed through Conda environments, and as such, is the only software that need be installed by the user.

- Conda (“Anaconda Software Distribution,” 2020) v23.7.2 (https://conda.io/projects/conda/en/latest/user-guide/install/download.html)
- Snakemake (Mölder et al., 2021) v7.16.0 (https://github.com/snakemake/snakemake/releases/tag/v7.16.0)
- minimap2 (Li, 2018) v2.24 (https://github.com/lh3/minimap2/releases/tag/v2.24)
- samtools (Danecek et al., 2021) vl.9 (https://github.com/samtools/samtools/releases/tag/1.9)
- SPADes (Bankevich et al., 2012) v3.15.2 (https://github.com/ablab/spades)
- BLAT (Kent, 2002) v20140318 (https://github.com/djhshih/blat/releases/tag/v35.1)
- Git (Spinellis, 2012) v2.30.1 (https://git-scm.com/)
- gdown (Wada, 2023) v4.7.1 (https://github.com/wkentaro/gdown/releases/tag/v4.7.1)
- Python (Python Software Foundation, 2023) v3.7–3.10

##### Sample File

Sample data is available in the TELCoMB GitHub repository in the /test_samples/samples/ directory. The included data, telcomb_sample.fastq, is a subset of reads from the human fecal sample reported by Slizovskiy et al. (2020). We will use the sample dataset to illustrate that each step in the pipeline functions as it should.

1. Install Conda if it is not already available. Conda can be installed using the installer from the website here https://www.anaconda.com/download/ or by using wget as follows:

wget -O conda.pkg \
https://repo.anaconda.com/archive/Anaconda3-2023.07-2-MacOSX-arm64.pkg
installer -pkg conda.pkg

  This example illustrates how to download and install the Anaconda package on an M-series MacBook; be sure to use the correct link for your device’s operating system.
2. Create the TELCoMB Conda environment using the following commands:

conda create -c conda-forge -c bioconda -n telcomb snakemake git
conda activate telcomb
git clone https://github.com/jonathan-bravo/TELCoMB.git
3. Set up a directory structure within the cloned GitHub repository.

mkdir -p telcomb/samples_dir/samples # for fasta files
4. The TELCoMB workflow assumes that all FASTQ files are stored in a target directory that contains a sample directory. Therefore, once all directories are created, link, copy or move the FASTQ files to this directory.
  Replace <data_dir> placeholder with the correct path.

# linking files to sample directory
for fq in <data_dir>/*.fastq; 
do
ln -s ${fq} telcomb/samples_dir/samples; # create symlink
done
5. Modify the following options in telcomb/config/config.json, depending on input/user preference. The default values are:

“WORKFLOW” :
{
“SAMPLEDIR” : “test_samples”,
“WORKDIR” : “test_dir”,
“DATABASES_DIR” : “databases”,
“LONG_READS” : “true”,
“DEDUPLICATE” : “true”
},

  The SAMPLEDIR value references the sample directory. The WORKDIR value references the directory where all output is placed. The initial value for SAMPLEDIR is set to test samples because of the included test input. DATABASE_DIR is the directory where the database files are downloaded. LONG_READS can be changed to false if the input data are short reads; otherwise, it should remain true. And finally, DEDUPLICATE can be set to false if the deduplication is causing the workflow to run beyond the desired time frame. It is highly recommended that this remains at the default value to improve quality of the results.
  Lastly, when running TELCoMB for the first time all databases and third-party software will be downloaded as part of the initial run of TELCoMB. For any subsequent run, data will only be generated for new samples that have been added or old samples that have been modified.

## ALTERNATE PROTOCOL

### INSTALLING TELCOMB ON A SLURM CLUSTER

Hardware, software, and file requirements are the same from Basic Protocol 1.

1. On a Slurm cluster, Conda can be loaded by running this command, if the Conda module is present on your cluster:

module load conda
2. Create the TELCoMB Conda environment using the following commands:

conda create -c conda-forge -c bioconda -n telcomb snakemake git
conda activate telcomb
git clone https://github.com/jonathan-bravo/TELCoMB.git
3. Set up a directory structure within the cloned GitHub repository, and we need to create an additional directory if the workflow is being run on a cluster.

mkdir -p telcomb/samples_dir/samples # for fasta files
mkdir -p telcomb/logs # for cluster log output
4. The TELCoMB workflow assumes that all FASTQ files are stored a target directory that contains a sample directory. Therefore, once all directories are created, we link, copy or move the FASTQ files to this directory. *Note: replace <data_dir> placeholder with the correct path*.

# linking files to sample directory
for fa in <data_dir>/*.fastq;
do
ln -s ${fa} telcomb/samples_dir/samples; # create symlink
done
5. Modify the run script and Slurm profile configuration file. First modify the telcomb/run.sh script to include the email and account information in the header. This informs the cluster manager which account to use to launch the initial snakemake command.

#SBATCH --job-name=TLS-disp
#SBATCH --account ≤account>
#SBATCH --qos ≤account qos>
#SBATCH --mail-type=FAIL
#SBATCH --mail-users≤email>
#SBATCH --ntasks=1
#SBATCH --cpus-per-task=1
#SBATCH --mem=1gb
#SBATCH --time=96:00:00
#SBATCH --output=logs/%j_disp.log
#SBATCH --error=logs/%j_disp.log

  Next, modify the telcomb/profiles/slurm/config.yaml file with the same account and email information. Additionally, provide or remove queue priority, or qos, information, which is typically provided by your account manager. This configuration JSON file is used by Snakemake to launch each individual rule with the specified settings, allowing Snakemake to work harmoniously with Slurm.

cluster:
mkdir -p logs/ &&
sbatch
--account={resources.account}
--qos={resources.qos}
--cpus-per-task=“1”
--nodes=l
--mem={resources.mem_mb}
--time=“72:00:00”
--job-name=“TLS-job”
--mail-user=“<email>“
--mail-type=“NONE”
--output=“logs/log_%j.log”
--error=“logs/err_%j.log”
default-resources:
- mem_mb=32000
- account≤account>
- qos≤qos>

  Also modify the following options in telcomb/config/config.json, depending on input/user preference. The default values are:

“WORKFLOW” :
{
“SAMPLEDIR” : “test_samples”,
“WORKDIR” : “test_dir”,
“DATABASES_DIR” : “databases”,
“LONG_READS” : “true”,
“DEDUPLICATE” : “true”
},

  The workflow value definitions can be found in Basic Protocol 1. Again, we highly recommend leaving the DEDUPLICATE default setting for higher quality results.
  When running TELCoMB for the first time, all databases and third-party software will be downloaded as part of the initial run. As in Basic Protocol 1, any subsequent run will only generate data for new samples that have been added or old samples that have been modified.

## BASIC PROTOCOL 2

### DATA PREPROCESSING

The preprocessing step prepares the input data for analysis. The target enrichment, protocol deploys PCR for DNA enrichment during adaptor-ligation and targeted site capture, which results in possible read duplication (Bansal, 2017). These duplicates need to be removed, otherwise the abundance will be incorrectly inflated. Hence, with long reads as input, the reads are subjected to a deduplication procedure using the following similarity-based approach. Reads are initially grouped by exact read length into individual bins, each read belonging to a single bin. These bins are then merged to make clusters of reads that are no more than 1.99–SIMILARITY THRESHOLD % longer than the shortest read length, each bin belonging to only a single cluster. This process creates a dynamic number of read clusters based on the number and length of reads present in the input data such that, on deduplication, no unnecessary computation is performed on reads that would never be considered duplicates because of length. Below is some pseudocode highlighting the process.


for read in reads:
get_read_length(read)
put_read_in_bin(read)
return bins
while bins:
cluster_max_len = ceil(bins[0]*1.09)
cluster = [x for × in bins if × ≤ cluster_max_len]
clusters.append(cluster)
bins = [x for × in bins if × not in cluster]
return clusters


All reads in every cluster are pairwise aligned with BLAT (Kent, 2002). Reads are considered duplicates if the span of all the hit/query High-scoring Segment Pairs were greater than or equal to the user-defined SIMILARITY_THRESHOLD, default at 90%, of the total hit/query length. Sets of duplicate reads are accumulated, and deduplicated FASTQ files are generated by randomly retaining a single read for each duplicated set from the original library FASTQ. For these analyses, the SeqIO module (Cock et al., 2009) from Biopython is used to parse FASTA/FASTQ files and Pattern Space Layout (PSL) files.


# across multiple threads 
for cluster in clusters:
psl = blat cluster cluster
make_dup_list_for_cluster(psl)
return dup_lists
dups = merge_dup_lists(dup_lists)
deduplicate(dups)


In addition to supporting long reads, targeted short reads can be used as input in TELCoMB. If short reads are used as input, they are assembled into contigs using MetaSPAdes (Nurk et al., 2017) before proceeding with the alignment to target databases. MetaSPAdes constructs a de Bruijn graph of all reads using SPAdes, simplifies that into an assembly graph, then traces paths along the assembly graph to form long genomic fragments within the metagenome (Bankevich et al., 2012; Nurk et al., 2017). Once we have the assembly from targeted short reads, we can proceed with the pipeline as though we have long-read input sequences. Supporting short reads increases accessibility of the pipeline to users that do not have access to methods for generating long reads. For long-read input, a read-lengths JSON file is generated at the start of the workflow, using the original FASTQ file before deduplication. However, for short-read input data, the read-lengths JSON is not generated until after the reads have been assembled into longer contigs.

1. If the data from the sequencer include multiple FASTQ files per sample, combine them using the cat command. This step assumes the files for each sample are in their own directory, i.e., all the files for sample 1 are in a directory named sample1 and all the files for sample 2 are in a directory named sample2.

cd <data_dir>/<sample_dir>
cat *.fastq>  . .<sample_id>.fastq
2. After concatenation, there should be a single FASTQ file for each of the input samples. Create symbolic links in the sample directory to these files from the source directory.

for fq in <data_dir>/*.fastq; 
do
ln -s ${fq} telcomb/sample_dir/samples; # create symlink
done

  FASTQ files can be copied to the sample directory, but linking the files reduces unnecessary use of hard drive space. The files can also be moved directly, but it is best not to operate directly on source files.
3. Review the settings in telcomb/config/config.json (Table 1), which can be adjusted to change input and output locations, how data is processed, and the threshold used for detecting duplicates.
4. Launch the workflow from the telcomb directory by either submitting the telcomb/run.sh script to the Slurm batch manager or by running the workflow locally. Using screen when connected to a server allows one to detach from the screen and allow the workflow to continue running if the remote session is interrupted.
  *If running the workflow locally, i.e., without a remote connection to a server/cluster, then the screen commands become completely optional*.
  To run locally, use the following command.

screen -S telcomb; #starting a screen with the name: telcomb
conda actiavte telcomb; #activating the conda environment
cd telcomb; #changing directories into telcomb
snakemake --cores <threads> --use-conda --latency-wait 20 \
--rerun-incomplete --conda-frontend conda #launching the workflow

  To run in a Slurm environment, submit the job using the sbatch.

cd telcomb
sbatch run.sh
5. Review the output files and figures, which are produced by this step in the workflow.
  *SN is the sample name and DS is the deduplicate string (i.e., deduplicated, not deduplicated, or assembled)*.
  - **Deduplicated, Assembled, or Non-Deduplicated Reads** (SN_DS): These are the reads or contigs that are produced from the selected deduplication method in telcomb/config/config.json, either produced by deduplicate, not_deduplicated_reads, or meta_spades_assembly; these reads or contigs are used as input for the alignments to the MGE and MEGARes databases.
  - **Read Lengths** (SN_read_lengths.json): A JSON formatted file produced by the read_lengths workflow rule that contains an entry for every input read identifier and the length value for that read. Length is determined by the number of base pairs in the read.
  - **Duplicate Reads** (SN.dup.reads.fastq): if the DEDUPLICATE setting in telcomb/config/config.json is set to true and the input data contain long reads, this FASTQ file is produced by the deduplicate workflow rule and contains all read entries from the input FASTQ file that were flagged as duplicates and removed.
  - **Read Length Plots** (SN_DS_read_lengths_hist.svg): This figure is produced by the read_length_plots workflow rule and showcases a bar chart displaying read lengths on the x-axis and their counts on the y-axis.

## BASIC PROTOCOL 3

### CALCULATION OF RESISTOME DISTRIBUTION AND COMPOSITION

After the reads are deduplicated or assembled, they are aligned to MEGARes v3 (Bonin et al., 2022) for ARG detection, and ACLAME v0.4 (Leplae et al., 2004), ICEberg v2.0 (Liu et al., 2019), and PlasmidFinder v2.1 (Carattoli et al., 2014) for MGE detection using Minimap2 (Li, 2018). MEGARes, now in its third version, features an acyclic ontology—meaning that each ARG can be unambiguously categorized by group, mechanism, and class. Given this, TELCoMB considers the ARGs on the accession, group, mechanism, and class levels since each of these are frequently reported. The counts for group, mechanism, and class are aggregated from the corresponding accessions. Similarly, TELCoMB reports each MGE as well as the number of MGE’s of every given type, i.e., plasmids, Integrative Conjugative Elements (ICEs), prophages, and virulence factors. The Sequence Alignment Map (SAM) files that resulted from alignment to the MEGARes and MGE databases are then used to characterize the resistomes and mobilomes in deduplicated reads or assembled contigs. Custom Python scripts were used to count the number of unique MEGARes classes, mechanisms, groups, and genes in each library. Next, the same script was used to count the number of unique MGE accessions by type.

Note that all the specified output is generated for each sample, while the heatmap and violin plots are generated for the entire dataset. Together, these files provide a detailed and multi-faceted view of the resistome and mobilome, crucial for understanding the complexity of antimicrobial resistance in mixed microbial populations.

The counts of unique features and alignments per feature were used to describe the richness and relative abundance of the resistome and mobilome features in each library. In the case of long reads, the counts correspond to the deduplicated read alignment, and in the case of short reads, the counts correspond to the alignment of the assembled contigs.

**Figure 2:**
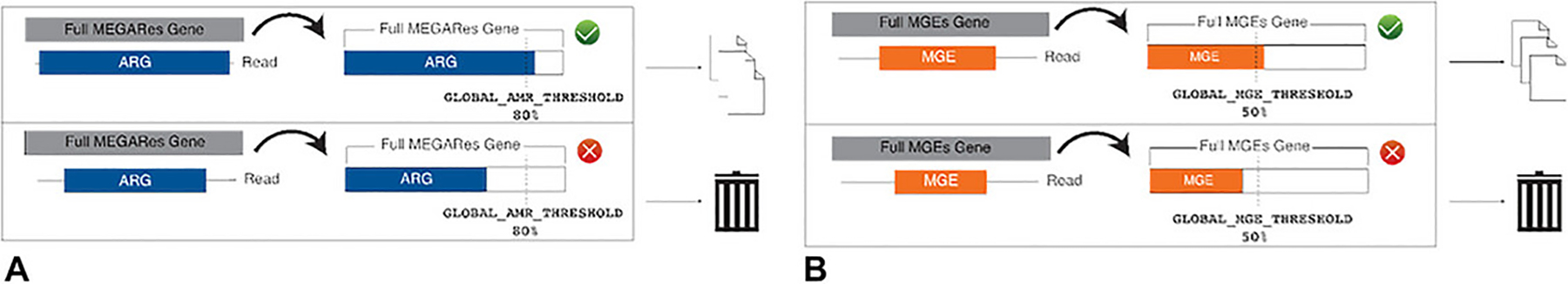
ARG and MGE Thresholds. ARGs and MGEs that aligned to the reads are only considered for colocalization, mobilome, and resistome analysis if the alignment covers the specified genomic fraction of the full gene in the target database. ARGs must have a default of 80% of the MEGARes gene covered by the alignment (*GLOBAL_AMR_THRESHOLD*), and MGEs must have a default of 50% of the MGEs gene covered by the alignment *(GLOBAL_MGE_THRESHOLD)*.

1. Review the parameter settings in the telcomb/config/config.json file found in Table 2. To reduce false-positive detection of ARG and MGE accessions, we used a gene fraction cutoff, defined as the proportion of nucleotides within a given reference accession that are aligned by at least one sequenced read of 80% and 50% for resistome and mobilome accessions, respectively, based on cut-offs established in a prior resistome-mobilome colocalization analysis of metagenomic data (Slizovskiy et al., 2022). These cut-offs are illustrated in Figure 2.
2. Launch the workflow, if not previously run or if configuration settings were changed since the last run, by navigating to the telcomb directory and either submitting the telcomb/run.sh script to the Slurm batch manager or running the workflow locally. Here is an example of running the workflow locally:

conda actiavte telcomb; #activating the conda environment
cd telcomb; #changing directories into telcomb
snakemake --cores <threads> --use-conda --latency-wait 20 \
--rerun-incomplete --conda-frontend conda #launching the workflow

**Figure 3:**
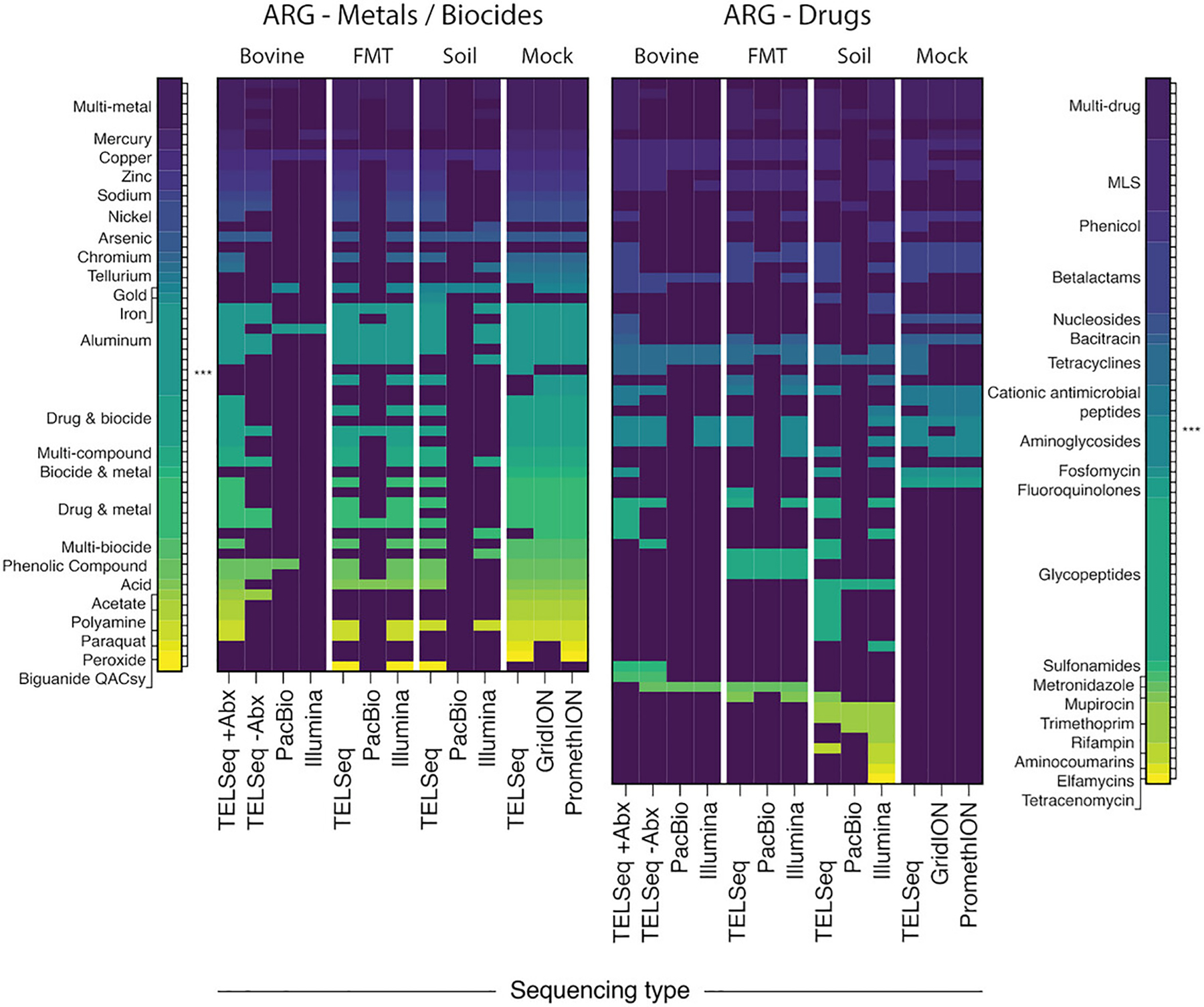
TELCoMB Heatmap. Binary heatmap of resistome composition at the ARG mechanism level, for metals and biocides (left) and antibiotic drugs (right), by sample type and sequencing platform. This image originates from Slizovskiy et al. (2020) and has been edited.

**Figure 4:**
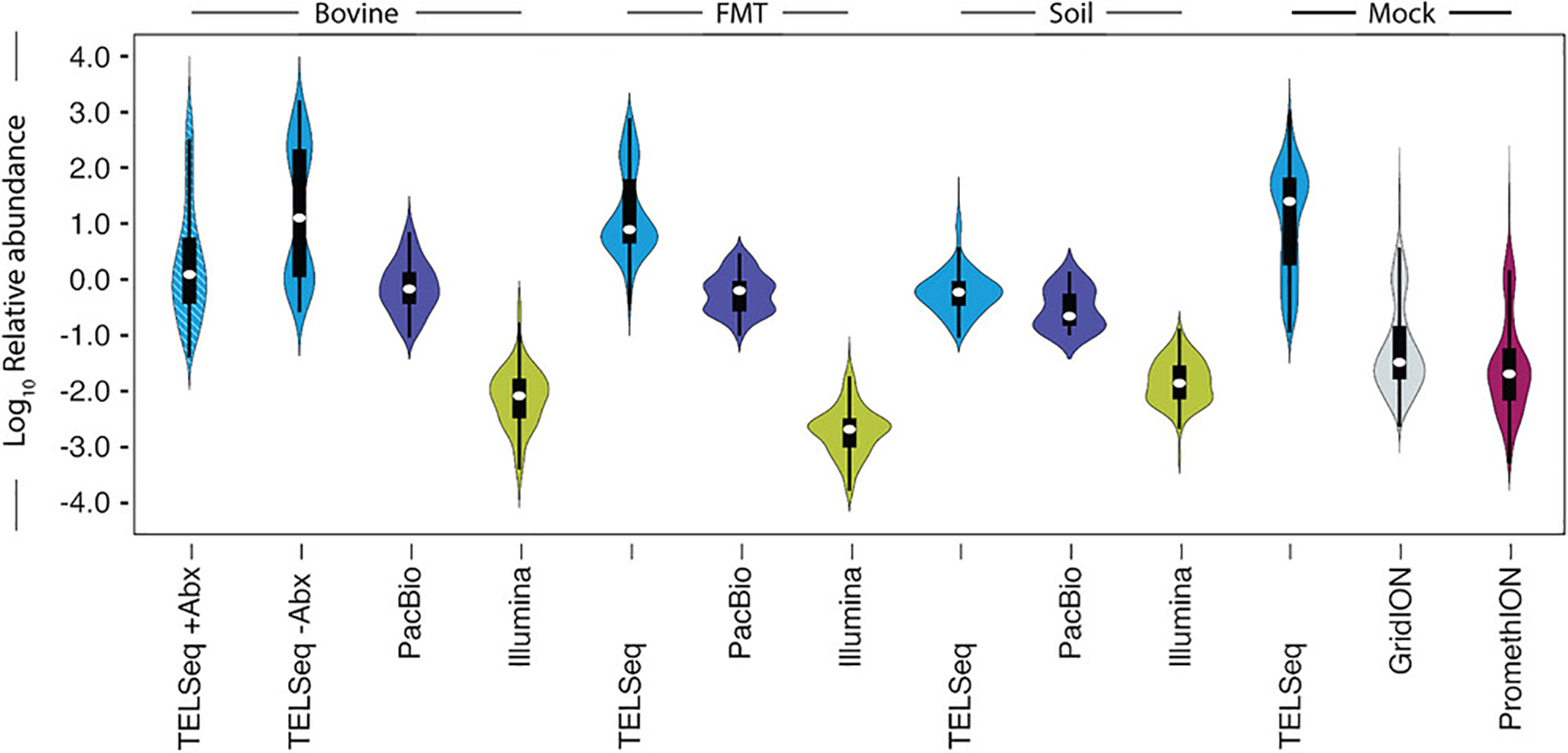
TELCoMB Violin Plot. Violin plots showing resistome distribution as the log10 relative abundance of ARG groups (y-axis), normalized for gene length and sequencing depth, by sample type and sequencing platform. This image originates from Slizovskiy et al. (2020).

  Here is an example of submitting the telcomb/run. sh script to the Slurm batch manager:

cd telcomb
sbatch run.sh
3. Review the output files. The following alignment files are produced:
  - **MEGARes Alignment** (SN_DS_ato_megares.sam): This SAM is obtained after aligning the deduplicated reads or assembled contigs to the MEGARes database. This SAM file contributed to generating the colocalization, resistome, and mobilome results.
  - **MGEs Alignment** (SN_DS_ato_MGES.sam): This is a SAM of the processed input data, either deduplication, assembly, or no deduplication, that is aligned to the MGEs database FASTA file produced by the align_to_mges workflow rule. Data from this alignment is used to generate colocalization, resistome, and mobilome results.
    Next, alignment files are used by the resistome and mobilome workflow rule to produce the Resistome Features, Resistome Richness, and Mobilome files:
  - **Resistome Features** (SN_DS.fastq_amr_features.csv): This is a CSV file that contains statistics on the number and lengths of reads or contigs. It provides detailed information for each MEGARes match, including MEGARes header, group, mechanism, and class, along with the number of supporting reads or contigs for each. The following statistics are given:
    - ARG_NUM_OF_READS: the number of reads or contigs that aligned to the MEGARes database
    - ARG_READ_LENGTH_MEAN: mean length of the reads or contigs aligned to the MEGARes database
    - ARG_READ_LENGTH_MEDIAN: median length of the reads or contigs aligned to the MEGARes database
    - ARG_READ_LENGTH_RANGE: a tuple with the shortest read length that aligned to the MEGARes database as the first value and the longest read length as the second value
    - ARG_READ_LENGTH_STD_DEV: standard deviation of the read lengths, closely related to the variance; a higher value means the read lengths are more spread out and a lower standard deviation means the read lengths are closer together
    - ARG_READ_LENGTH_VARIANCE: variance of the read length; a high value indicates that the read lengths are spread far from the mean and a low value indicates that the read lengths are closer together
    - ARG_READ_LENGTH_SKEW: the measure of asymmetry of the read length distribution, a positive value indicating a right skew, and a negative value indicating a left skew
    - ARG_READ_LENGTH_KURTOSIS: kurtosis is a measure of the shape of the distribution of the length of reads or contigs; a positive number indicates that the distribution has fatter tails and a sharper peak at the mean or a higher likelihood of extreme values; a negative number indicates thinner tails and a flatter peak or fewer extreme values, and a value of zero indicates that the distribution is similar to normal.
    - ARG descriptors. The following descriptors are given for each ARG alignment.
      - MEGARes identifier: the MEGARes identifier for the ARG. A detailed description of the MEGARes identifiers can be found on the MEGARes website. https://www.meglab.org/.
      - Num Reads: the number of reads or contigs that aligned to the ARG
      - Group: the MEGARes group of the ARG
      - Num Reads: the number of reads or contigs that aligned to ARGs classified within each group
      - Mechanism: the MEGARes mechanism of the ARG
      - Num Reads: the number of reads or contigs that aligned to ARGs classified within each mechanism
      - Class: the MEGARes class of the ARG
      - Num Reads: the number of reads or contigs that aligned to ARGs within each class.
    - **Resistome Richness** (SN_DS.fastq_amr_richness.csv): This CSV file reveals the diversity within the resistome by showing counts of different genes, gene classes, mechanisms, and groups. The column headers are:
      - Gene Richness: the count of unique ARGs for each sample;
      - Class Richness: the count of unique ARG classes for each sample;
      - Mechanism Richness: the count of unique ARG mechanisms for each sample;
      - Group Richness: the count of unique ARG groups for each sample.
    - **Mobilome** (SN_DS.fastq_mobilome.csv): This CSV file offers statistics about the number and length of reads or contigs that aligned to accessions within any of the reference MGE databases. The following statistics are given:
      - MGES_NUM_OF_READS: number of reads or contigs that aligned to the MGE databases
      - MGES_READ_LENGTH_MEAN: mean length of the reads or contigs aligned to the MGE databases
      - MGES_READ_LENGTH_MEDIAN: median length of the reads or contigs aligned to the MGE databases
      - MGES_READ_LENGTH_RANGE: a tuple with the shortest read length that aligned to the MGE databases as the first value and the longest read length as the second value
      - MGES_READ_LENGTH_STD_DEV: standard deviation of the read lengths, closely related to the variance, a higher value means the read lengths are more spread out and a lower standard deviation means the read lengths are closer together
      - MGES_READ_LENGTH_VARIANCE: variance of the read length, a high value indicates that the read lengths are spread far from the mean and a low value indicates that the read lengths are closer together
      - MGES_READ_LENGTH_SKEW: the measure of asymmetry of the read length distribution, a positive value indicating a right skew, and a negative value indicating a left skew
      - MGES_READ_LENGTH_KURTOSIS: kurtosis is a measure of the shape of the distribution of the length of reads or contigs, a positive number indicates that the distribution has fatter tails and a sharper peak at the mean or a higher likelihood of extreme values, while a negative number indicates thinner tails and a flatter peak or fewer extreme values, and a value of zero indicates that the distribution is similar to normal
      - MGE Richness: number of unique MGE alignments (diversity)
      - MGE descriptors. The following descriptors are given for each MGE accession: MGE accession header (obtained from the FASTA file), and the number of reads or contigs that aligned to the relevant MGE.
  In addition to these statistics, TELCoMB gives the same statistics when reads or contigs with whose length is four standard deviations or move from the mean are removed.
4. Review the output figures. Each figure uses data from Slizovskiy et al. (2020) and is an example of the output that can be expected from TELCoMB. A heatmap (heatmap_all_samples.svg) is produced by the heatmap workflow rule. It illustrates the resistome composition at the ARG mechanism level for all samples (one sample per column), with one panel for metal and biocide ARGs and a separate panel for antibiotic drug ARGs (Fig. 3). A violin plot (violing_plot_all_samples.svg) is produced by the violin_plots workflow rule and represents the resistome distribution of all samples as the *log* 10 relative abundance of ARG groups on the y-axis (Fig. 4). Note that the relative abundance is normalized by the length of the ARG, and the total length of all the reads in the sample.

## BASIC PROTOCOL 4

### IDENTIFICATION OF ARG-MGE COLOCALIZATIONS

The MEGARes database alignment file and the MGE database alignment file are parsed with a custom script to generate a CSV file in which each row contains the following colocalization information: deduplicated read or assembled contig identifier, MEGARes database accession and MGE database accession, and position of the genes on the read. Note that each possible combination of ARG and MGE gene is considered and stored in the CSV file. Only unique colocalizations are reported. Colocalizations are defined as non-unique if the following conditions are true: 1) the ARGs belong to the same MEGARes group; 2) the MGEs have the same database accession; and 3) the colocalization distance (defined as the distance between the end of the ARG alignment on the read and the start of the MGE alignment or vice versa) between the colocalizations are within 250 bp of each other. A custom Python script is used to group colocalizations into 250-bp intervals based on the order in which they were encountered going through the CSV of colocalizations. For example, if three colocalizations with the same MEGAResgroup and MGE accession were encountered with colocalization distances of 50 bp, 150 bp, and 350 bp, in that order, the first two colocalizations would be considered non-unique and the third would be considered unique.

Lastly, in order to identify high-confidence colocalizations and reduce false positives, a deduplicated read or an assembled contig must contain both an ARG and an MGE at 80% and 50% gene fraction, respectively, again based on previously used thresholds. We apply convergence restrictions for all identified ARGs, removing the MGE sequence if there is an overlap between the ARG and MGE covering at least 50% of the smallest sequence, removing the potential for multiple alignments to both the ARG and MGEs within the same start:stop position on the read. This was done due to extensive sequence homology between multiple accessions in the ARG and MGE reference databases.

**Figure 5:**
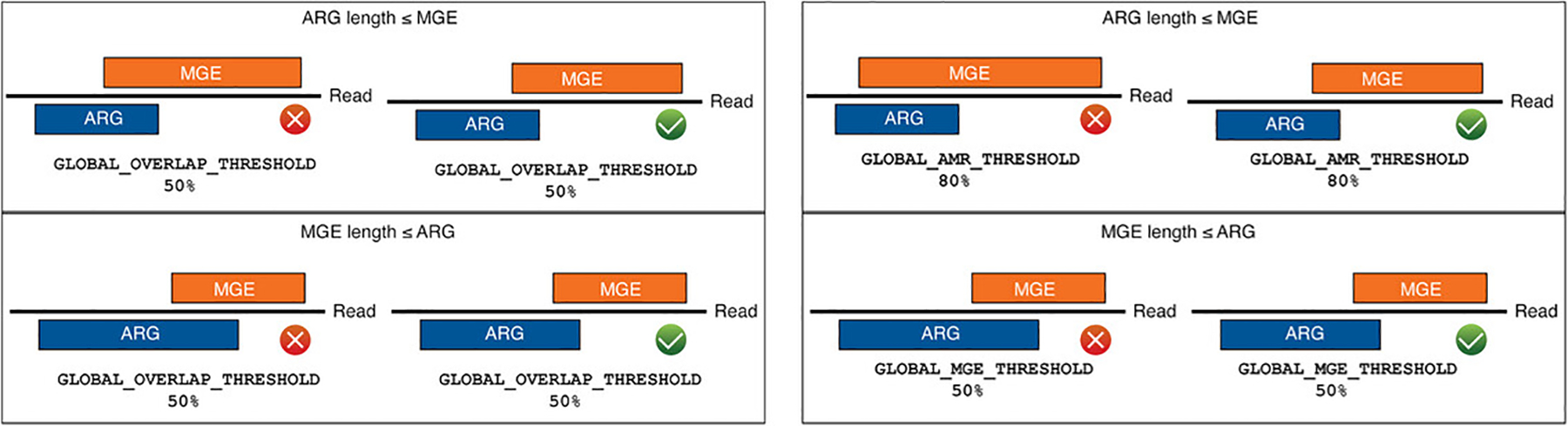
Overlap Strategies. The figure on the left illustrates the use of the *GLOBAL_OVERLAP_THRESHOLD* to determine if a MGE will be considered invalid due to offending overlap, if the *OVERLAP_THRESHOLD_STRATEGY* is set to ONE. The figure on the right demonstrates the use of the *GLOBAL_AMR_THRESHOLD* when an ARG is shorter than an MGE, and *GLOBAL_MGE_THRESHOLD* when an MGE is shorter than an ARG, to determine if a MGE will be considered invalid due to offending overlap if the *OVERLAP_THRESHOLD_STRATEGY* is set to *TWO*.

1. Review the parameter settings in the telcomb/config/config.json file (Table 3). Two parameters affect the colocalizations. Figure 5 illustrates the difference between colocalization strategies.
2. Launch the workflow, if not previously run or if configuration settings were changed since the last run, by navigating to the telcomb directory and either submitting the telcomb/run.sh script to the Slurm batch manager or running the workflow locally. Here is an example of running the workflow locally:

conda actiavte telcomb; #activating the conda environment
cd telcomb; #changing directories into telcomb
snakemake --cores <threads> --use-conda --latency-wait 20 \
--rerun-incomplete --conda-frontend conda #launching the workflow

**Figure 6:**
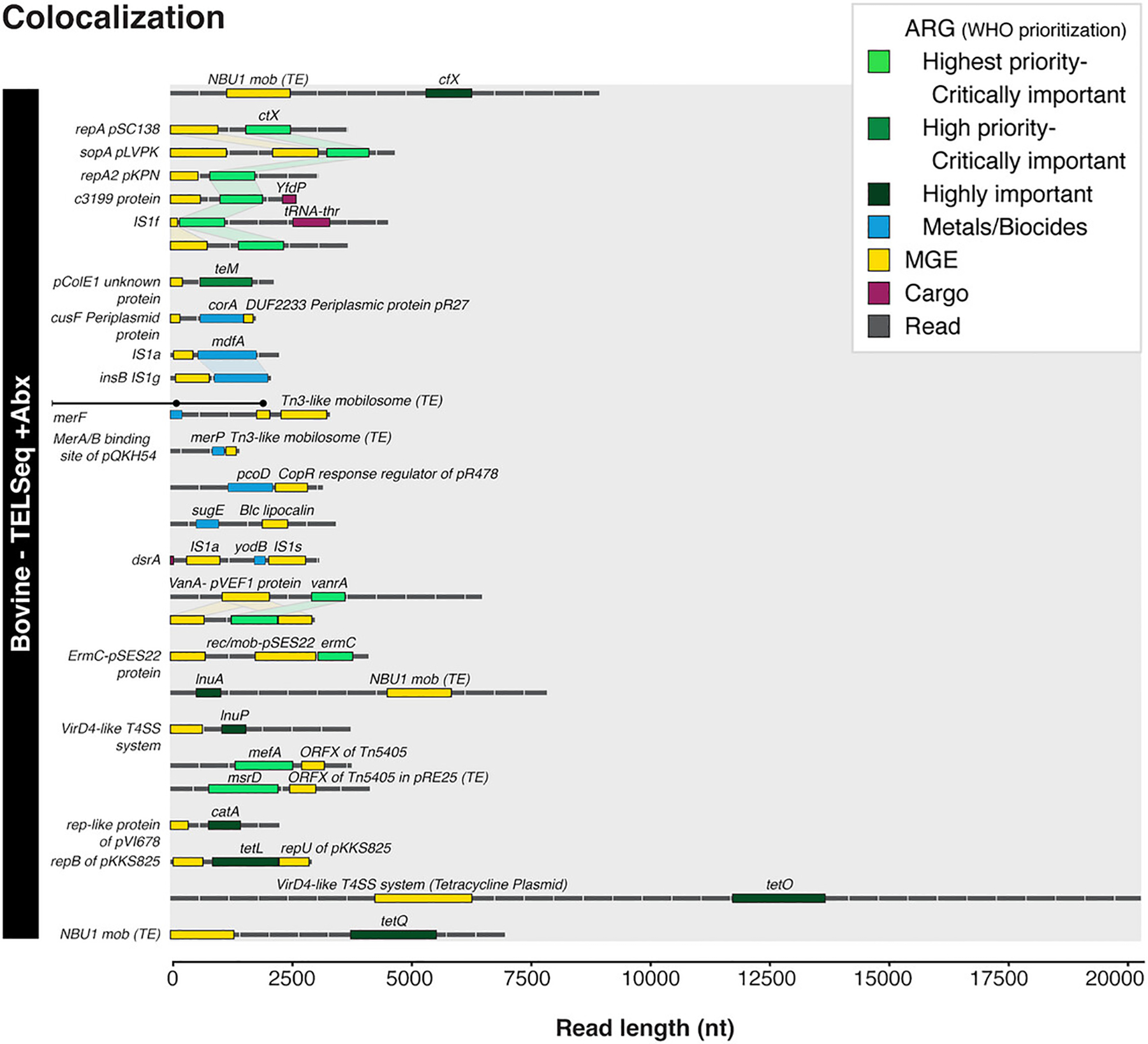
ARG-MGE Colocalizations on Reads or Contigs. Individual TELCoMB reads or contigs (black horizontal dashed lines, length on x-axis) containing both ARGs (green and blue) and MGEs (yellow), separated by sample type. The ARG colors indicate the World Health Organization’s (WHO) classification status. Light green: Highest priority, critically important; Medium green: High priority, critically important; Dark green: Highly important. This image is a composite image of multiple samples’ colocalizations and comes from the previous work of Slizovskiy et al. (2020). The workflow produces a simpler image per sample.

  Here is an example of submitting the telcomb/run.sh script to the Slurm batch manager:

cd telcomb
sbatch run.sh
3. Review the output files. The first file is the Overlapping MGEs file produced by the overlap workflow rule:
  - **Overlapping MGEs** (SN_DS_overlapped_mges.csv): This CSV file contains the MGE headers of MGEs that overlap with ARGs beyond the specified threshold. Offending MGEs are then listed in this file and not used when generating colocalization or mobilome results.
    *This file affects the mobilome results from* Basic Protocol 3
    The next two files are the Colocalizations and Genes List files, which are produced by the find_colocalizations workflow rule:
  - **Colocalizations** (SN_DS.fastq_colocalizations.csv): This CSV file presents a row for each ARG-MGE colocalization. The default configuration settings specify that an ARG has 80% genome fraction, a MGE that has at least 50% genome fraction, these occur together on a single read (or contig), and any gene overlaps that occur are evaluated and offending MGEs are removed if they cross the default threshold of 50%. This file gives the read or contig name, gene names, and gene positions on the read or contig. The column headers are:
    - read: the header value for the read from the deduplicated FASTQ file or contig id from the assembly FASTQ file
    - ARG: the header from MEGARes indicating the accession to which the read aligned
    - ARG positions: the start:stop positions of the ARG on the read
    - MGE (s): the header from the MGE databases indicating the accession to which the read aligned
    - MGE (s) positions: the start:stop positions of the MGE on the read
  - **Genes List** (SN_DS_genes_list.xlsx): This Excel file contains a row for every read containing an ARG. This is an intermediate file generated while looking for candidate colocalizations. The column headers are:
    - Read Name: The read id from the input SAM file
    - AMR Genes: A semicolon-separated list of ARG headers obtained from the alignment to the MEGARes database
    - AMR Gene Pos: A semicolon-separated list of ARG start:stop positions from the alignment to the MEGARes database
    - MGE Genes: A semicolon-separated list of MGE gene headers from the alignment to the MGEs database
    - MGE Genes Pos: A semicolon-separated list of MGE gene start:stop positions from the alignment to the MGEs database.
    The Colocalization Richness file is produced from the Colocalizations file by the colocalization richness workflow rule:
  - **Colocalization Richness** (SN_DS.fastq_colocalization_richness.csv): This CSV file includes a row for each unique colocalization that contains both gene names, the distance between them, and the number of times this colocalization occurs in the data. There is also a count value for the number of unique colocalizations (i.e., colocalization richness) at the top of the file. The column headers are:
    - MEGARes group: the gene group name(s)
    - MGE gene: the MGEs database header for the MGE accession(s)
    - Distance: the distance between the end of the ARG or MGE and the start of the other gene within the colocalization
    - Occurrences: the number of occurrences the colocalizations appear based on the ARG and MGE alignments
4. Review the output figures. The workflow produces an illustration of the colocalizations (SN_colocalizations_plot.svg) via the colocalization_visualization workflow rule. It illustrates each read containing an ARG-MGE colocalization for each sample, with reads or contigs depicted as black lines and genes as colored rectangles (Fig. 6).

## COMMENTARY

### Background Information

**Figure 7:**
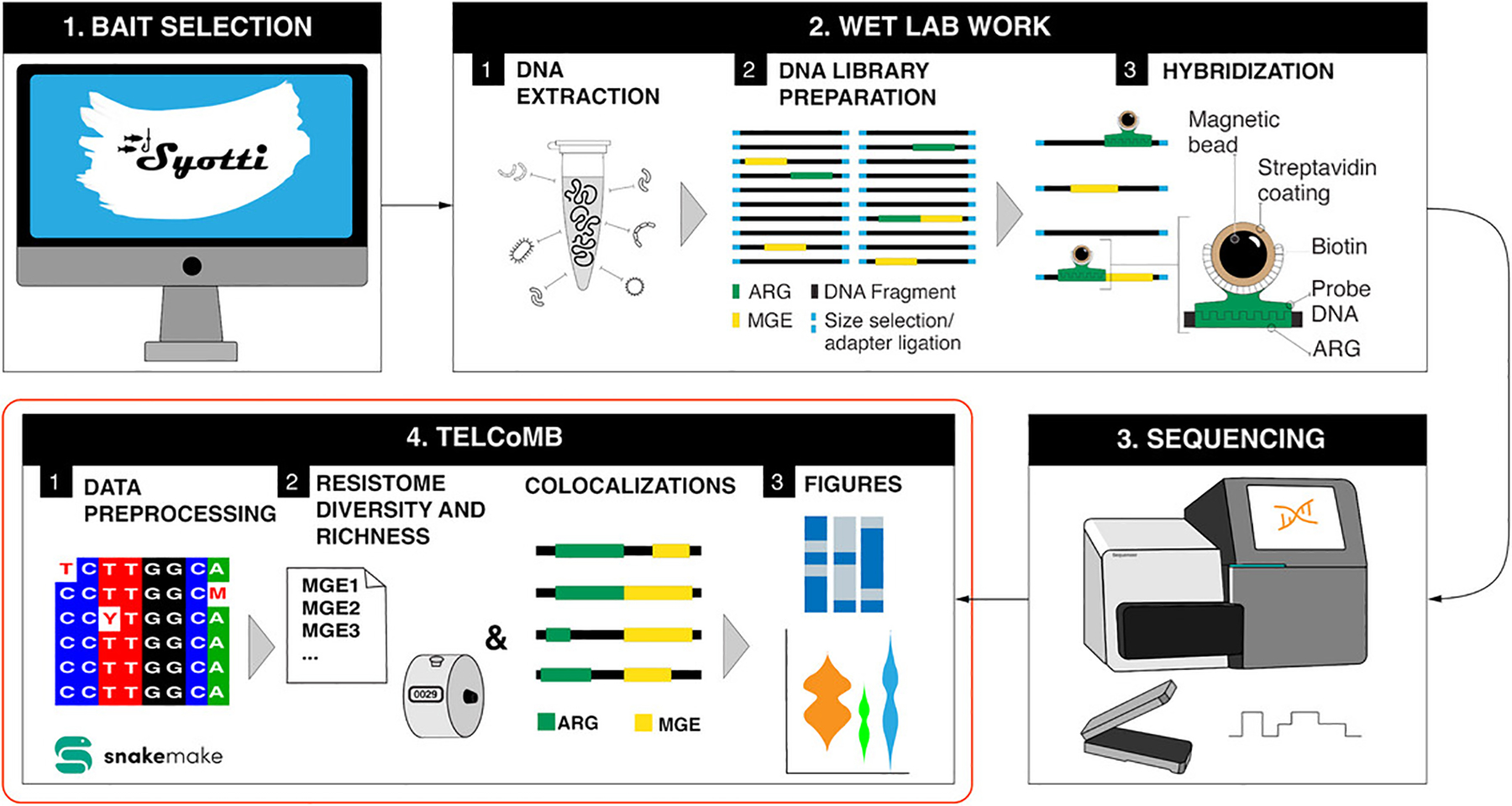
TELCoMB Overview. The software package (1) Syotti is used for in silico bait design. In the wet lab, (2.1) DNA extraction is performed. The DNA then undergoes size selection, purification, and (2.2) library preparation, followed by (2.3) hybridization with custom-designed biotinylated 120-mer probes. After the samples undergo capture and amplification they are (3) submitted for sequencing. The resulting reads are then run through TELCoMB, starting with (4.1) data preprocessing, where reads are deduplicated or assembled, reads and read lengths are counted, and the reads are aligned to MEGARes and the MGEs databases. (4.2) The resulting SAM files are then used to generate mobilome, resistome, and colocalization results. Finally the mobilome, resistome, and colocalization results are used for (4.3) figure generation.

Here we present a workflow for analyzing data that has been enriched via cRNA biotinylated probes for ARGs. ARGs encompass a small portion of a metagenomic sample due to their molecular size and abundance. Thus, cRNA biotinylated probes and other enrichment techniques can greatly enhance the sensitivity and specificity of sequencing methods for antimicrobial resistance studies. These probes are complementary RNA molecules tagged with biotin, designed to hybridize with specific ARG sequences in a complex nucleic acid mixture. However, prior to sequencing, the probes are created *in silico* by software such as Syotti (Alanko et al., 2022), which takes as input a set of genomic targets in the form of a FASTA file, and a parameter defining the length of the probes, and outputs a set of DNA probes that can be used for enrichment. The output of Syotti is sent to a manufacturer to generate synthetic probes, which hybridize to the target DNA in the sample. The ARG-bound biotinylated probes are then captured using magnetic beads, enriching the sample for ARG-containing sequences. The enrichment protocol has been combined with both short-read (Lee et al., 2017; Noyes et al., 2017) and long-read sequencing (Slizovskiy et al., 2020). We illustrate the process in Figure 7. By enriching samples for ARGs, cRNA biotinylated probes significantly improve the detection of low-abundance ARGs and enhance the overall quality of sequencing data, contributing to a better understanding of AMR and microbial genetics. Lastly, although TELCoMB was designed for ARG-MGE colocalization, we believe its use stretches beyond this specific use case and can be broadened for the analysis of other genetic targets in complex microbial species. For example, the identification of plant pathogens, viral strains and sub-strains, Internal Transcribed Spacer (ITS) regions, and the colocalization within them.

### Critical Parameters

The parameters, contained in telcomb/config/config.json, given in Table 4 affect the resources made available to the tools run during the workflow. We note that each of the parameters has a default setting that is given in the table, and that if any resource settings are changed in telcomb/config/config.json and the workflow is being run on a cluster, then the associated values should also be changed in the telcomb/profiles/slurm/config.yaml file for applicable rules.

### Troubleshooting

In the event of an error while running the workflow, Snakemake will generate a log file stored in the telcomb/logs directory if running using a Slurm managed cluster or in the telcomb/.snakemake/log/directory. As dot (.) files are usually hidden by the operating system, you will have to go into the file explorer settings and allow viewing of hidden items to see this directory. Errors in the log will be labeled Error.

Snakemake also has built-in support for forcing individual rules to execute by using the -R flag. This is useful if a rule has changed, but you do not want to run the whole pipeline from that step and instead want to do some error checking and ensure the data generated here is correct before letting the pipeline re-run the rest of the following processes. Example:

Snakemake --cores <threads> --use-conda
--latency-wait 20 \
-R find_colocalizations --conda-frontend conda
#launching the workflow


Snakemake also allows you to re-run the entire workflow in place, in the event that some files need to be regenerated. If files are removed, the workflow can be relaunched and Snakemake should be able to run only the steps of the workflow that are needed to finish generating the required output.

Some troubleshooting advice can be found in Table 5. Additional issues with the workflow can be posted to the Issues tab on Github repository.

### Timing

**Figure 8:**
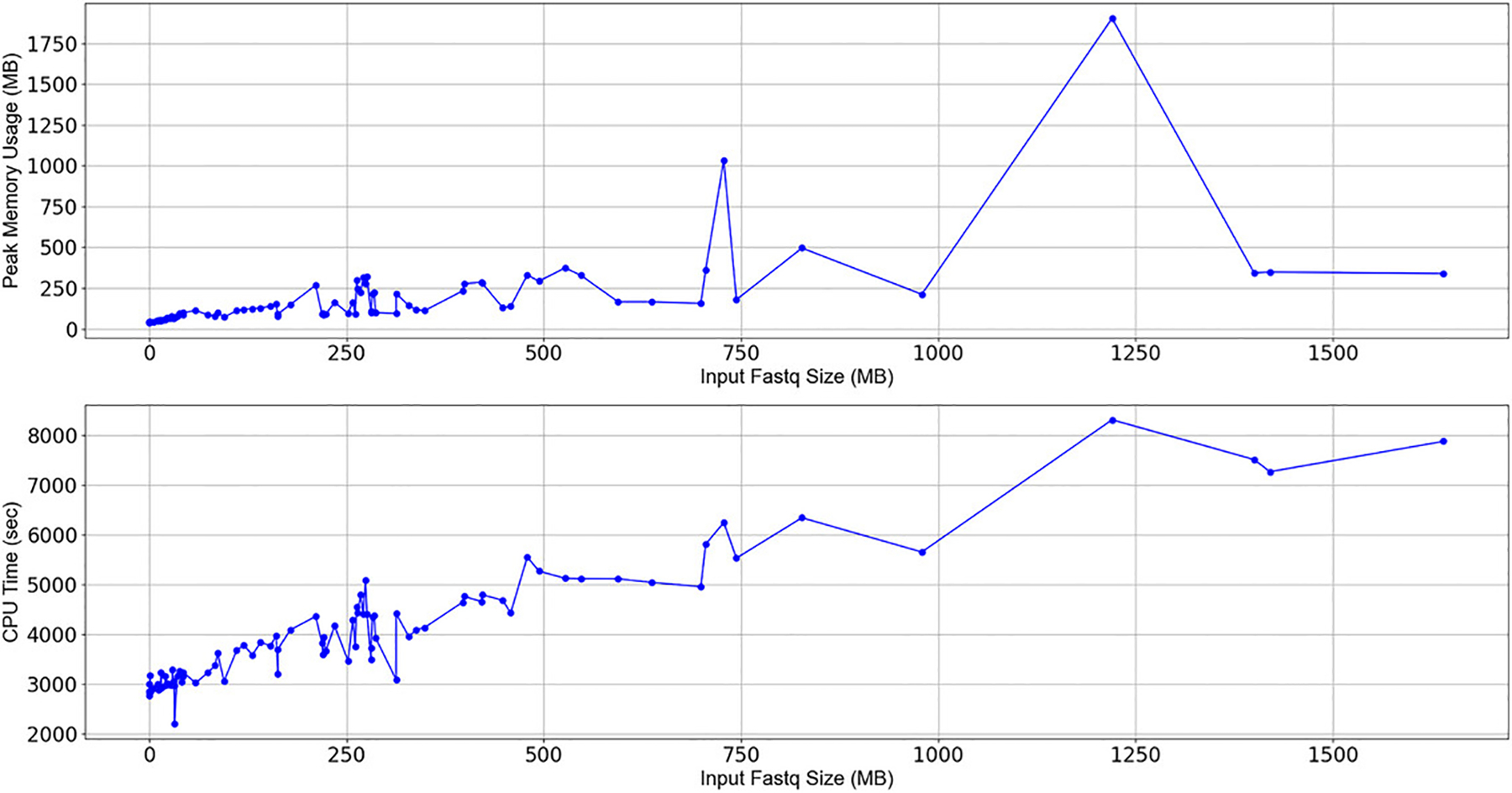
Resource Plots. The top graph shows memory use as a function of sample size and the bottom graph shows CPU time as a function of sample size. Values were achieved using the default *slurm* profile.

Running TELCoMB on our sample dataset required less than three minutes of CPU time. We then ran each sample from Slizovskiy et al. (2020) individually and collected memory and CPU usage metrics using the built-in benchmark feature of snakemake (Fig. 8). The relationship between sample size and resource usage is not always linear, given different numbers of colocalizations and different alignments in different samples. Keep in mind that this graph shows single samples running through the workflow when, generally, multiple samples are processed simultaneously.

A few steps in the workflow will, by default, request more than 1 CPU core and will require more than 8 GB of memory to function efficiently:

- At the start of the workflow, if long reads are used, all pairwise alignments are identified for deduplication. A custom python script will launch multiple instances of BLAT, allowing the workflow to process many read clusters in parallel. The number of BLAT instances launched is determined by the number of CPU cores passed as either an argument in the command line or the value specified in the configuration file DEFAULT:32.
- If input data are short reads, then instead of deduplication, SPADes will be used to perform assembly, so reads can be processed by the rest of the workflow without additional changes. By default, the k-mer flag for SPADes is set to DEFAULT: 21,33,55,77,127 to ensure that a decent assembly is achieved. However, this k-mer setting will require a larger amount of memory to process, so the default value for memory is set at DEFAULT:128 GB. There is also a DEFAULT: 32 CPU cores specified for SPADes because of the size complexity of the k-mer selection.
- Finally, both alignment steps to the MEGARes and MGEs databases use Minimap2 with a DEFAULT: 12 CPU cores requested. This value can be lowered much more easily than the other values. Note that having additional CPU cores for these alignment steps has led to a non-insignificant reduction in run time.

If fewer threads are specified using the -c flag when launching the Snakemake workflow, then all jobs are limited by default to using that number of threads, regardless of the number of specified threads in the configuration file. If, however, the number of threads specified when launching the workflow far exceeds the number of threads specified in the configuration file, the workflow can use the default values specified and can run many of the steps in parallel.

## Figures and Tables

**Table 1:** Basic Protocol 2 Parameters. Values in telcomb/config/config.json that Inform the Workflow of the Input, Input and Output Locations, and How to Preprocess the Input Data

Parameter	Definition
SAMPLEDIR	The path to the directory that contains your samples directory [string]. The default is test_samples.
WORKDIR	The name of the directory that will contain all of your output [string]. The default is test_dir.
DATABASE_DIR	The name of the directory that the database files will be downloaded to, [string]. The default is databases.
LONG_READS	A flag denoting weather or not the input data is long reads or short reads [true, false]. The default is true.
DEDUPLICATE	A flag denoting if input reads should be deduplicated [true, false]. The default is true.
DEDUPLICATION_SIMILARITY_THRESHOLD	A value that sets the percentage of similarity required from both the query sequence and the target sequence, used when finding duplicates [float]. The default is 0.9.

**Table 2:** Basic Protocol 3 Parameters. The Threshold Values Used to Determine if the Proper Gene Fraction has been Reached to Include the Gene in Further Analysis

Config parameter	Definition
GLOBAL_AMR_THRESHOLD	Value that determines the genome fraction required for an ARG alignment to be included in colocalization, and resistome analysis, the value is also used as an overlap cutoff when OVERLAP_THRESHOLD_STRATEGY is set to TWO [float]. The default is 0.8.
GLOBAL_MGE_THRESHOLD	Value that determines the genome fraction required for an MGE alignment to be included in colocalization, and mobilome analysis, the value is also used as an overlap cutoff when OVERLAP_THRESHOLD_STRATEGY is set to TWO [float]. The default is 0.5.

**Table 3:** Basic Protocol 4 Parameters. These Parameters Affect the Overlap Detection Between ARGs and MGEs

Parameter	Definition
GLOBAL_THRESHOLD_STRATEGY	This flag determines the overlap strategy to use. ONE uses the OVERLAP_THREASHOLD for both the ARG and MGE overlaps. TWO uses GLOBAL_MGE_THRESHOLD for MGE overlaps and GLOBAL_AMR_THRESHOLD for ARG overlaps. The default is ONE.
OVERLAP_THRESHOLD	Threshold that determines if there is a disqualifying overlap of a MGE with an ARG, the set value determines the percentage of the MGE or ARG that must be overlapping for the offending MGE to be excluded, this value is used if OVERLAP_THRESHOLD_STRATEGY is set to ONE [float]. The default is 0.5.

**Table 4:** Application Parameters. Settings for the Assembly and Alignment Tools

Application	Parameter	Definition
Minimap2	ALIGNER_OPTIONS	Alignment parameters that should be used for PacBio data. These parameters can be changed to include any valid Minimap2 flags but it is recommended to use either -ax map-pd or -ax map-ont, the -a flag should be included because this flag generates the CIGAR string required for sample processing after alignment [string]. The default is -ax map-pb.
	THREADS	The number of CPUs that will be made available to Minimap2 [int]. The default is 12.
BLAT	THREADS	The number of CPUs that will be made available to BLAT [int]. The default is 32.
SPAdes	THREADS	The number of CPUs that will be made available to SPAdes [int]. The default is 32.
	MEMORY	The amount of system memory in gigabytes that will be made available to SPAdes [int]. The default is 128.
	PHRED	PHRED quality offset for the input reads [33, 64]. The default is 33.
	KMERS	A comma-separated list of k-mer sizes to be used by the assembler. All values must be odd, less than 128 and listed in ascending order [list [int]]. The default is 21, 33, 55, 77, 127.

These settings should be changed according to both hardware limits and data processing needs. Default settings are recommended for accuracy and speed of processing.

**Table 5:** Troubleshooting Advice: Potentially Common Issues That May Occur When Initially Setting Up the Workflow, the Possible Reason for the Problem, and the Solution for That Problem

Problem	Possible reason	Solution
Conda will not initialize	Corrupted Conda installation	Try to reinstall Conda through the Anaconda web page, or try a different installation (mamba)
The telcomb environment will not create or initialize	The packages being installed could have version mismatches that collide	Specify the version of the programs after looking up the available versions in Conda
The workflow fails to complete	The directory structure could be incorrect	Make sure that the correct directories are present and the configuration file is correct
Permission denied error on a script	Scripts not executable	Make sure that all scripts have the correct permissions using chmod +x workflow/scripts/*
Conda frontend error	Mamba not installed	make sure to include the flag -- conda - frontend conda, this can be a bit slower than mamba but all environments run as they should
Memory errors	SPADes flag is set too high	Make sure to edit the config [“ SPADES”] [“MEMORY”] value to match the available resources
Storage errors	Intermediate files too large	Intermediate PSL files generated during deduplication might take up a large amount of space depending on the input read count
Output is incorrect	Samples files might not all be linked	Make sure all sample files are directly copied using cp or linked using ln -s

## Data Availability

The reference database and annotation files for MEGARes v3.0 are available at https://meglab.org, for PlasmidFinder v2.1 https://bitbucket.org/genomicepidemiology/plasmidfinder db, for ACLAME v0.4 http://aclame.ulb.ac.be/, for ICEberg v2.0 https://bioinfo-mml.sjtu.edu.cn/ICEberg2. The test sample used in the procedure is available in the TELCoMB GitHub repository at https://github.com/jonathan-bravo/TELCoMB/tree/main.
